# Thrombophlébite cérébrale: complication d’une cellulite fronto-temporo pariétale chez un enfant victime d’une morsure humaine

**DOI:** 10.11604/pamj.2020.35.14.15776

**Published:** 2020-01-16

**Authors:** Soufiane Badsi, Soufiane Diyas, Mohamed Aabdi, Brahim Housni

**Affiliations:** 1 Service de Réanimation, CHU Mohamed VI, Faculté de Médecine et de Pharmacie d’Oujda, Université Mohammed 1^er^ Oujda, Oujda, Maroc

**Keywords:** Thrombophlébite cérébrale, enfant, trouble de conscience, anti-coagulation, Cerebral thrombophlebitis, child, conscience disorder, anticoagulation

## Abstract

La thrombophlébite cérébrale est considérée comme une pathologie rare mais grave dont la fréquence sous-estimée est de plus en plus reconnu chez les enfants et contrairement aux adultes la prise en charge reste controversée. Nous rapportons le cas d’un enfant de 12 ans ayant été victime d’une morsure par un ami au niveau du cuir chevelu de la région fronto-temporale de la tête, évoluant par l’apparition d’une tuméfaction évoquant une cellulite fronto-temporo pariétale compliquée d’une thrombophlébite cérébrale, diagnostiquée sur la présentation clinique et l’imagerie. Notre patient était admis dans notre structure dans un tableau de trouble de conscience, ainsi le recours aux anticoagulants et au traitement étiologique a permis une bonne évolution clinique.

## Abstract (en)

Cerebral thrombophlebitis is a rare but severe disease whose underestimated frequency is increasingly recognized among children and, unlike adults, management is controversial. We report the case of a 12-year old child bitten by a friend at the level of the scalp, in the frontotemporal region of the head. This bite evolved due to the occurrence of a swelling evoking a frontotemporoparietal cellulitis complicated by cerebral thrombophlebitis, diagnosed based on the clinical feature and imaging. The patient was admitted in our hospital in a state of conscience disorder. Anticoagulant and etiological treatments enabled good clinical evolution.

## Introduction

La thrombophlébite cérébrale est considérée comme une pathologie rare mais grave [1-3] dont la fréquence sous-estimée est de plus en plus reconnu chez les enfants [4] et contrairement aux adultes, la prise en charge reste controversée. Le progrès et l’accessibilité de l’imagerie cérébrale permettent actuellement un diagnostic précoce de la thrombose veineuse cérébrale (TVC) et donc le début rapide de l’enquête étiologique, dont découlent le pronostic et le traitement spécifique amenant le praticien dans certains cas à effectuer une prophylaxie anti thrombotique de façon systématique chez les enfants particulièrement exposés.

## Patient et observation

Nous rapportons le cas d’un enfant âgé de 12 ans ayant été victime d’une morsure par un ami, au cours d’une bagarre de rue, au niveau du cuir chevelu de la région fronto temporale de la tête. L’enfant avait bénéficié chez soi de soins locaux de la plaie et une automédication faite par un antalgique simple et un anti-inflammatoire. L’évolution était marquée par l’apparition d’un œdème des parties molles de la région lésée et d’une fièvre rebelle au traitement symptomatique qui se sont aggravés au cours du temps (20 jours). Par la suite il avait installé un trouble de conscience motivant ainsi la famille à s’adresser aux urgences de notre centre hospitalier pour éventuelle prise en charge. A l’admission, la conscience était altérée avec un score de Glasgow à 10. L’examen clinique avait révélé une tuméfaction de consistance molle de la région fronto temporale droite du cuir chevelu. L’examen ophtalmologique avait objectivé un œdème papillaire stade 1 du même côté. L’interrogatoire des parents n’a retrouvé aucun antécédent familial de thrombose. La tomodensitométrie et l’angio-imagerie par résonance magnétique (IRM) ont mis en évidence une collection hypodense de la région fronto-temporo pariétale droite étendue jusqu’aux parties molles palpébrales droites mesurant 68/19mm et étendu sur 55mm avec thrombose du sinus longitudinal supérieur.

Le bilan biologique se présentait comme suit: globules blancs: 14 650mm^3^; protéine C-Réactive (CRP): 65; procalcitonine: 1,8; hémoglobine: 11,9; plaquettes: 226 000mm^3^; taux de prothrombine: 81%; temps de céphaline activée (TCA): normal; la fonction hépatique et rénale étaient normales; les profils de coagulation étaient tous normaux. La ponction lombaire était réalisée et la méningite était exclue. Le diagnostic d’une cellulite était posé et une antibiothérapie probabiliste faite de céphalosporine (Triaxon), puis adapté à l’antibiogramme, était débutée complétée par un drainage de la région infectée. Un traitement par de l’énoxaparine (lovenox) avait été institué. L’enfant avait également été mis sous oxygénothérapie, antalgique et corticothérapie. L’évolution était marquée par l’amélioration de l’état clinique et biologique de l’enfant après 25 jours d’hospitalisation.

## Discussion

La TVC est rare et touche particulièrement l’enfant. Une publication d’une grande série canadienne du registre canadien des accidents vasculaires cérébraux a estimée l’incidence de la TVC chez les enfants à 0,67 pour 100 000 enfants [4]. Les troubles de l’hémostase et les anomalies pariétales constituent les principaux mécanismes physiopathologiques à l’origine de la thrombophlébite cérébrale. Des anomalies de la coagulation sont observées dans près de 20% des thromboses veineuses cérébrales [2, 3, 5]. Rosenstingl *et al*. [3] recommandaient donc une étude systématique de l’hémostase après toute thrombophlébite cérébrale. Chez notre patient, la recherche d’anomalie constitutionnelle de l’hémostase n’a pas été faite. Les manifestations cliniques de la TVC ne sont pas spécifiques et peuvent être subtiles [6] principalement chez l’enfant [7]. La majorité des cas de TVC se présentent avec des symptômes d’une hypertension intracrânienne et des signes neurologiques focaux [8]. Notre patient avait présenté un trouble de conscience avec un œdème papillaire. Une attention particulière devrait être accordée à ce type de symptomatologie imposant la réalisation d’exploration neuroradiologique dans les plus brefs délais.

L’étiologie de la thrombophlébite cérébrale peut être multiple [3] et les causes infectieuses sont de loin les plus fréquentes. Cette constatation souligne l’intérêt d’entreprendre des mesures préventives. En effet, Les thromboses veineuses d’origine septique ont considérablement diminué depuis l’introduction des antibiotiques [9] et dans la majorité des séries récentes cette cause n’est plus prédominante. Chez notre patient, l’origine septique était la cause de la TVC. L’imagerie cérébrale joue un rôle primordial dans le diagnostic positif de la TVC. Si le scanner permet de poser le diagnostic de thrombose veineuse cérébrale, l’IRM, notamment l’angio-IRM est actuellement l’examen de choix [1, 2]. L’angio-IRM permet de visualiser la thrombose et de suivre son évolution, sous forme d’isosignal en T1 à la phase aigue, puis d’hypersignal persistant, notamment en T2. Notre patient a bénéficié initialement d’une tomodensitométrie injectée puis complété par une angio-IRM et le diagnostic d’une thrombophlébite du sinus longitudinal supérieur était posé.

L’instauration rapide d’un traitement basé sur l’anticoagulation avec de l’héparine par voie parentérale a permis une nette amélioration du pronostic vital et fonctionnel des enfants [3] et c’est d’ailleurs ce qu’ont prouvé plusieurs études [5]. En effet, l’héparinothérapie permet la perméabilité vasculaire, l’atténuation de la croissance et propagation du thrombus et le retour à la normal de l’examen neurologique [8]. Le traitement thrombolytique est généralement réservé aux cas sévères avec un risque élevé de mortalité. Dans notre cas, un traitement précoce par l’anticoagulation a donné de bons résultats et le malade était déclaré sortant, sans déficit neurologique, après 25 jours de traitement. Nous n’avons pas pu suivre notre malade plus qu’un mois et donc on ne sait pas s’il avait présenté des événements récurrents.

## Conclusion

La thrombophlébite cérébrale de l’enfant est rare. Les mesures préventives s’avèrent nécessaires dans cette population pour diminuer l’incidence de cette affection grave. L’enquête étiologique devrait être complète et systématique et une prise en charge bien codifiée permet d’améliorer le pronostic des malades.
